# Pentacyclic Triterpenoid Acids Inhibit the Expression of Quorum Sensing-Related Virulence Factors and the Formation of Biofilm in *Pseudomonas aeruginosa* PAO1

**DOI:** 10.3390/antibiotics15060623

**Published:** 2026-06-20

**Authors:** Tsiry Rasamiravaka, Adeline Mol, Pierre Duez, Mondher El Jaziri, Marie Baucher

**Affiliations:** 1 Laboratory of Biotechnology and Microbiology, University of Antananarivo, Antananarivo 101, Madagascar; 2 Laboratory of Plant Biotechnology, Université Libre de Bruxelles, 6041 Gosselies, Belgium; adeline.mol@ulb.be (A.M.); mondher.el.jaziri@ulb.be (M.E.J.); marie.baucher@ulb.be (M.B.); 3 Unit of Therapeutic Chemistry and Pharmacognosy, University of Mons, 7000 Mons, Belgium; pierre.duez@umons.ac.be

**Keywords:** biofilm, *Pseudomonas aeruginosa*, quorum sensing

## Abstract

**Background/Objectives**: Numerous natural compounds have been reported to exhibit anti-virulence properties against pathogenic bacteria. Particularly, plants constitute a rich source of anti-quorum-sensing (QS) and anti-biofilm compounds with highly diverse chemical structures. Notably, several studies reported that plant-derived pentacyclic triterpenoids exert anti-biofilm activity against *Pseudomonas aeruginosa* without affecting bacterial viability, suggesting that this class of naturally occurring chemical compounds may represent a source of potent and clinically relevant anti-biofilm agents. **Methods**: To further investigate this hypothesis, we evaluated several commercially available pentacyclic triterpenoid acids of the oleanane, ursane and lupane types for their potential impact on QS mechanisms and biofilm formation in the *P. aeruginosa* PAO1 model strain. **Results**: Oleanane-type (oleanolic acid and maslinic acid), ursane-type (ursolic acid and corosolic acid) and lupane-type (betulinic acid) triterpenoids inhibited the expression of the QS-regulated *lasB* and *rhlA* genes as well as biofilm formation, without affecting bacterial growth. Among tested compounds, oleanolic and ursolic acids, at 400 µM, exhibited the strongest anti-biofilm activities, with 45% and 40% inhibition, respectively. Fluorescence microscopy revealed a marked disorganization of biofilm architectures, with bacterial communities failing to establish compact cell-to-cell attachment and confluent microcolonies. Further analyses indicated that these triterpenoid acids did not affect the expression of QS-regulator genes (*lasR/I* and *rhlR/I*), suggesting that their impact on *lasB* and *rhlA* expression and biofilm formation is independent of the *las* and *rhl* systems. **Conclusions**: These findings suggest that oleanane and ursane triterpenoid acids represent promising chemical backbones for the development of strategies aimed at inhibiting *P. aeruginosa* biofilm formation.

## 1. Introduction

In the ongoing fight against bacterial infections and in response to the widespread emergence of antibiotic-resistant bacteria, research on alternative or complementary strategies to the use of antibiotics has gained increasing attention [1]. One of the promising approaches, called “anti-virulence”, aims to disrupt the bacterial ability to colonize host tissues and cause damage [2]. Over the past two decades, anti-virulence strategies have been increasingly investigated, particularly in inherently multidrug-resistant bacteria such as *P. aeruginosa*, an opportunistic pathogen that mainly affects severely immunocompromised patients [3].

*P. aeruginosa* virulence factors are mainly coordinated by quorum-sensing (QS) mechanisms, a cell-to-cell communication system that enables bacteria to sense their population density through the production and detection of diffusible signal molecules that synchronize collective behaviours [4]. In this bacterium, two major QS systems, *las* and *rhl*, are particularly well characterized. In both systems, expression of *lasI* and *rhlI* genes drives the production of signal molecules acyl-homoserine lactones (AHLs) N-(3-oxododecanoyl)-L-homoserine lactone (3-oxo-C12-HSL) and N-butanoyl-L-homoserine lactone (C4-HSL), which are perceived by the transcriptional activators LasR (encoded by *lasR* genes) and RhlR (encoded by *rhlR* genes), respectively [5]. At high cell density, LasR and RhlR bind their respective signal molecules, increasing *lasI* and *rhlI* expression while triggering the production of several virulence factors, including LasB elastase, LasA protease, exotoxin A, rhamnolipids, pyocyanin, and cytotoxic lectins [6,7].

The *las* [8,9] and *rhl* [10,11] systems also regulate biofilm formation, a bacterial lifestyle in which communities of bacteria are embedded within a matrix of extracellular polymeric substances (EPS) that mediate cohesion between cells and adhesion to surfaces [12]. These EPS are essential for biofilm formation and architecture [13], and they confer protection against external aggressions, notably phagocytosis, host responses and antibiotics [14]. *P. aeruginosa* produces at least three types of EPS: (*i*) alginate, a linear polysaccharide composed of β-1,4-linked L-guluronic and D-mannuronic acids [15]; (*ii*) Pel, a glucose-rich polysaccharide, with unclarified composition; and (*iii*) Psl, a repeating pentasaccharide consisting of D-mannose, L-rhamnose, and D-glucose. As a “nonmucoid” *P. aeruginosa* strain, the PAO1 strain, isolated from an infected wound [16], produces low levels of alginate at the expense of Pel and Psl exopolysaccharides, which are considered to be critical for biofilm formation and maintenance [17]. In addition, the second messenger bis-(3′-5′)-cyclic dimeric guanosine monophosphate (c-di-GMP) is known to control the bacterial transition between planktonic and biofilm lifestyles [18].

**Figure 1:**
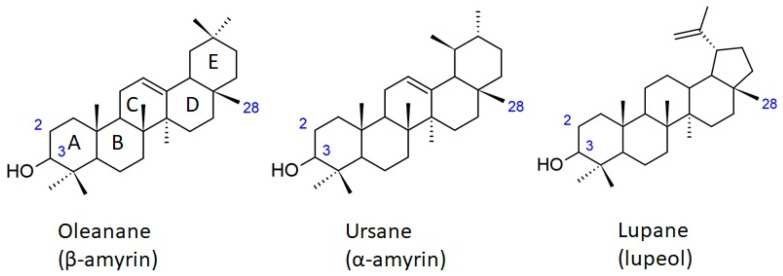
Skeleton of three main bioactive pentacyclic triterpenoids, oleanane-, ursane- and lupane-types, with their five rings (A, B, C, D and E). The biosynthesis of these pentacyclic triterpenoids is initiated with the cyclization of 2,3-oxidosqualene, leading to the generation of triterpenol, which presents a hydroxyl at position C-3 (β-amyrin for oleananes, α-amyrin for ursanes and lupeol for lupanes). Oxidation of β-amyrin, α-amyrin and lupeol at the C-28 position and hydroxyl addition at the C-2 position leads to various triterpenoids widely distributed in plants that exhibit important biological activities.

Natural compounds with highly diverse chemical structures, including alkaloids, organosulfurs, phenolics and terpenoids have been widely reported to exhibit antibacterial activity against pathogenic bacteria [3,19]. For instance, the alkaloids berberine and hordenine, isolated from *Coptis chinensis* Franch and *Hordeum vulgare* L., respectively, exhibit significant anti-biofilm activities against *P. aeruginosa* [20]. Likewise, the flavones baicalein and wogonin from *Scutellaria baicalensis* Georgi significantly reduce *S. aureus* biofilm formation, whereas the flavanones naringenin and taxifolin reduced biofilm formation and the production of QS-controlled virulence factors in *P. aeruginosa* PAO1 [20,21]. Among terpenoids, the pentacyclic triterpenoids, which constitute a large class of natural products characterized by a five-ring skeleton (Figure 1), have also attracted considerable interest due to their antimicrobial and anti-virulence effects [22]. Interestingly, the lupane-type triterpenoids betulinic acid and betulin significantly attenuated the production of QS-regulated virulence factors and biofilm formation in *P. aeruginosa* PAO1 [23]. Likewise, oleanolic and ursolic acids, the most common aglycones in the oleanane- and ursane-type triterpenoids, respectively, have been shown to inhibit biofilm formation by pathogenic bacteria. For instance, oleanolic acid from *Vernonia auriculifera* Hiern reduces biofilm formation of Gram-negative bacteria such as *Klebsiella pneumoniae* and *P. aeruginosa* (35% and 45% inhibition, respectively) at sub-minimum inhibitory concentrations (sub-MIC) (<550 µM and <2.2 mM, respectively) [24]. Similarly, ursolic acid (at 22 µM) from *Diospyros dendo* Welw. ex Hiern inhibits *Escherichia coli* and *P. aeruginosa* biofilm formation (79% and 87% inhibition, respectively) without affecting bacterial growth [25]. Recently, α-amyrin, a biosynthetic precursor of ursolic acid isolated from *Platostoma rotundifolium* (Briq.) A. J. Paton (Lamiaceae) was shown to exert anti-biofilm properties against *P. aeruginosa* PAO1 at 200 µM without affecting bacterial viability [26]. Based on a previous study and Kiplimo et al. reports [24], we arbitrarily used oleanolic acid at 800 µM as a positive control in the anti-biofilm assay, which exerted a similar inhibition level to α-amyrin at the same concentration. Taken together, these data suggest that the pentacyclic triterpenoid class may represent a promising source of anti-biofilm compounds that deserve to be explored in depth, particularly in terms of biological activity spectrum and structure-activity relationship. Thus, the present study investigates the potential of commercially available oleanane, ursane and lupane triterpenoids as anti-QS and anti-biofilm compounds against *P. aeruginosa* PAO1. It further explores their possible mechanism of action as well as the influence of chemical substitutions at the C-2 and C-28 positions.

## 2. Results

### 2.1. Oleananes, Ursanes and Lupanes Inhibit the Expression of the lasB and rhlA Genes in a Dose-Dependent Manner

**Figure 2:**
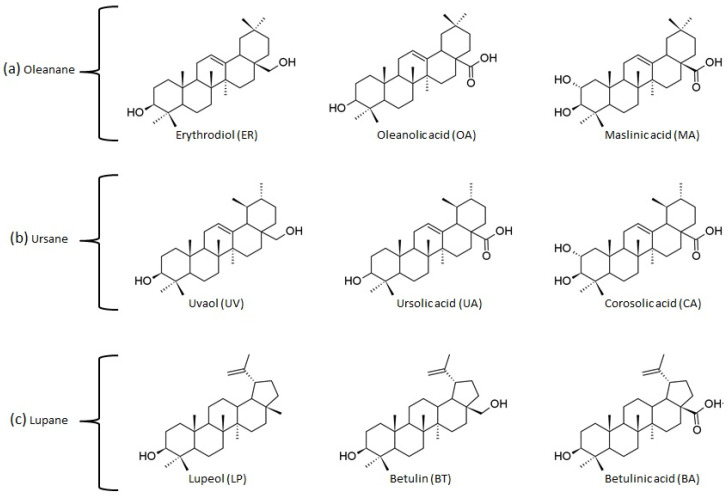
Chemical structures of tested triterpenoids: (**a**) Oleananes, erythrodiol [(3β)-olean-12-ene-3,28-diol; ER], oleanolic acid [(3β)-hydroxyolean-12-en-28-oic acid; OA] and maslinic acid [(2α, 3β)-dihydroxyolean-12-en-28-oic acid; MA]. (**b**) Ursanes, uvaol [(3β)-urs-12-ene-3,28-diol; UV], ursolic acid [(3β)-hydroxyurs-12-en-28-oic acid; UA] and corosolic acid [(2α, 3β)-dihydroxyurs-12-en-28-oic acid; CA]. (**c**) Lupanes, lupeol [(3β)-lup-20(29)-en-3-ol; LP], betulin [(3β)-lup-20(29)-ene-3,28-diol; BT] and betulinic acid [(3β)-hydroxylup-20(29)-en-28-oic acid; BA].

For the screening of anti-QS activity, three oleananes, erythrodiol (ER), oleanolic acid (OA) and maslinic acid (MA), three ursanes, uvaol (UV), ursolic acid (UA) and corosolic acid (CA), and three lupanes, lupeol (LP), betulin (BT) and betulinic acid (BA) (Figure 2) were evaluated at concentrations ranging from 100 to 800 µM for their effects on the expression of two QS-regulated genes, *lasB* (encoding LasB elastase) and *rhlA* [encoding 3-(3-hydroxyalkanoyloxy) alkanoic acids required for the production of rhamnolipids]. Concentrations higher than 800 µM were not tested due to a difficulty in solubilizing certain compounds in the DMSO solvent, particularly OA. Naringenin was selected as the reference compound for its proven anti-QS/anti-biofilm properties against PAO1 at 4000 µM [27,28].

**Figure 3:**
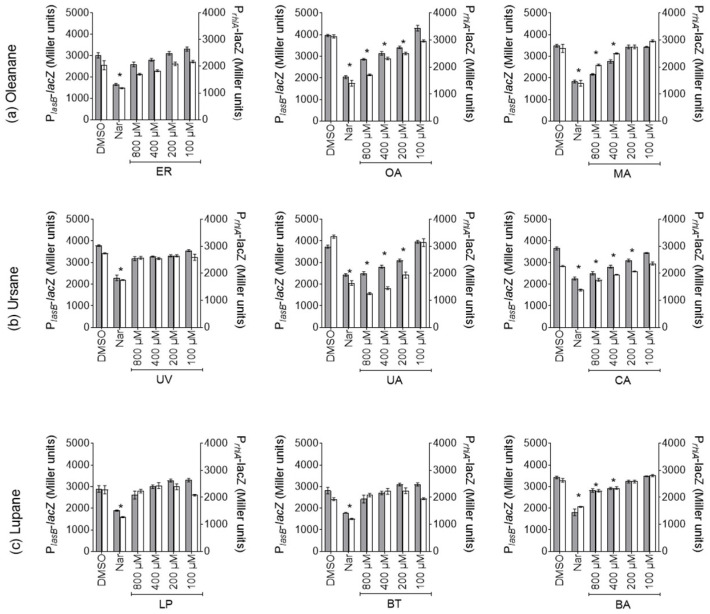
Effect of triterpenoids on QS genes (*lasB* and *rhlA*) expression in *P. aeruginosa* PAO1: (**a**) Effect of oleananes [erythrodiol (ER), oleanolic acid (OA) and maslinic acid (MA)] on *lasB* (grey bar) and *rhlA* (white bar) expression following 18 h of growth. (**b**) Effect of ursanes [uvaol (UV), ursolic acid (UA) and corosolic acid (CA)] on *lasB* (grey bar) and *rhlA* (white bar) expression following 18 h of growth. (**c**) Effect of lupanes [lupeol (LP), betulin (BT) and betulinic acid (BA)] on *lasB* (grey bar) and *rhlA* (white bar) expression following 18 h of growth. Triterpenoids were tested at different concentrations (from 100 to 800 µM). Naringenin (Nar, 4000 µM) is used as a reference QS inhibitor and dimethylsulfoxide (DMSO, 1%) as a solvent control. To estimate gene expression, absorbance of β-galactosidase activity was measured at 420 nm and expressed in Miller units. All experiments were conducted following five technical replicates and three independent biological replicates. Asterisks indicate samples that are significantly different from the DMSO control (*p* < 0.01) and error bars represent the standard errors of the means.

The data summarized in Figure 3 show that, among oleananes, OA and MA at 800 µM inhibit the expression of *lasB* (by 30% and 35%, respectively) and *rhlA* (by 60% and 25%, respectively), whereas ER had no effect on their expression. These inhibitory effects were dose-dependent. OA, but not MA, still reduced *lasB* and *rhlA* expression by 15% and 22%, respectively, at 200 µM but had no effect at 100 µM.

Similarly, among ursanes, UA and CA but not UV inhibited the expression of *lasB* (by 40% and 30%, respectively) and *rhlA* (by 70% and 25%, respectively). These inhibitory effects were dose-dependent. At 200µM, both UA and CA still reduced *lasB* and *rhlA* gene expression (by 55% and 30% for UA and by 25% and 16% for CA, respectively), whereas neither compound was effective at 100 µM.

In contrast, among lupanes, only BA exhibited a weak inhibitory effect on *lasB* (17% inhibition) and *rhlA* (14% inhibition) expression at 400 and 800 µM, whereas BT and LP were inactive.

**Figure 4:**
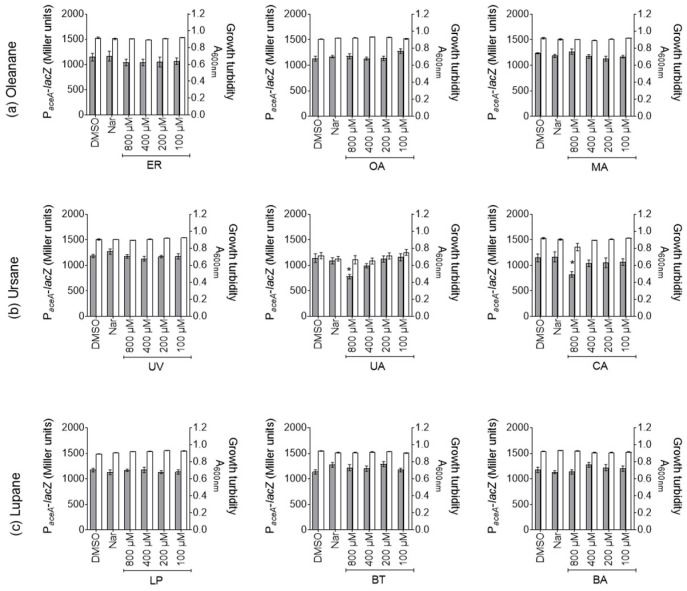
Effect of triterpenoids on *aceA* expression in *P. aeruginosa* PAO1: (**a**) Effect of oleananes [erythrodiol (ER), oleanolic acid (OA) and maslinic acid (MA)] on *aceA* expression (grey bar) and bacterial turbidity (white bar) following 18 h of growth. (**b**) Effect of ursanes [uvaol (UV), ursolic acid (UA) and corosolic acid (CA)] on *aceA* expression (grey bar) and bacterial turbidity (white bar) following 18 h of growth. (**c**) Effect of lupanes [lupeol (LP), betulin (BT) and betulinic acid (BT)] on *aceA* expression (grey bar) and bacterial turbidity (white bar) following 18 h of growth. Triterpenoids are tested at different concentrations (from 100 to 800 µM). Naringenin (Nar) at 4000 µM is used as a reference QS inhibitor and DMSO 1% as a solvent control. To estimate gene expression, absorbance of β-galactosidase activity was measured at 420 nm and expressed in Miller units. Growth turbidity was measured as A_600nm_. All experiments were conducted following five technical replicates and three independent biological replicates. Asterisks indicate samples that are significantly different from the DMSO control (*p* < 0.01) and error bars represent the standard errors of the means.

As shown in Figure 4, none of the triterpenoids (at 800 µM) adversely affected bacterial growth, as assessed by A_600nm_ and colony-forming unit (CFU) counts (Table S1), indicating that bacterial viability was not impaired. To clarify whether, as previously reported [27,28,29], the reduction in β-galactosidase activity was not due to effects on the transcription/translation mechanisms, we also measured the impact of these compounds on the QS-independent gene *aceA* (encoding an isocitrate lyase) expression. Ursanes (UA and CA) reduced the *aceA* expression only at 800 µM (by 32% and 25%, respectively; see Figure S1). This suggests that ursane acids may affect QS-related gene expression through effects on housekeeping functions of *P. aeruginosa* PAO1 or on β-galactosidase enzyme activity. As no effect on *aceA* expression was observed at lower concentrations of triterpenoids, 400 µM was selected for all further experiments.

### 2.2. Oleanane, Ursane and Lupane Triterpenoid Acids Do Not Affect the Expression of QS Regulator Genes in P. aeruginosa PAO1

**Figure 5:**
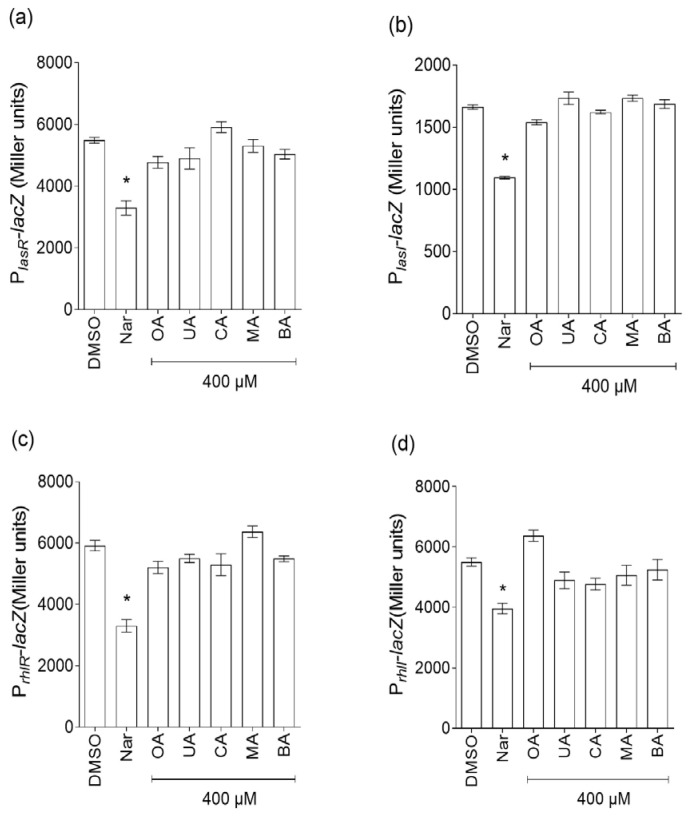
Effect of triterpenoid acids on QS genes (*lasR/I* and *rhlR/I*) expression in *P. aeruginosa* PAO1. (**a**) Effect of triterpenoid acids on *lasR* gene expression following 18 h of growth. (**b**) Effect of triterpenoid acids on *lasI* gene expression following 18 h of growth. (**c**) Effect of triterpenoid acids on *rhlR* gene expression following 18 h of growth. (**d**) Effect of triterpenoid acids on *rhlI* gene expression following 18 h of growth. All triterpenoid acids [oleanolic acid (OA), maslinic (MA), ursolic (UA), corosolic (CA) and betulinic (BA) acids] were tested at 400 µM. Naringenin (Nar, 4000 µM) is used as a reference QS inhibitor and dimethylsulfoxide (DMSO, 1%) as a solvent control. To estimate gene expression, absorbance of β-galactosidase activity was measured at 420 nm and expressed in Miller units. All experiments were conducted following five technical replicates and three independent biological replicates. Asterisks indicate samples that are significantly different from the DMSO control (*p* < 0.01) and error bars represent the standard errors of the means.

Because the tested triterpenoids at 400 µM impaired the QS-regulated (*lasB* and *rhlA*) gene expression in *P. aeruginosa* PAO1, their effects on QS systems (*lasR/I* and *rhlR/I*) were further evaluated in the same model. Specifically, the expression of the AHL synthetase genes *lasI* and *rhlI* and the QS regulator genes *lasR* and *rhlR* was evaluated following treatment with these triterpenoids at 400 µM. The results highlight that the tested oleananes, ursanes and lupanes did not significantly affect *lasR/I* and *rhlR/I* QS systems (Figure 5), suggesting that the inhibition of *rhlA* and *lasB* expression is not directly linked to the *las* and *rhl* QS systems.

### 2.3. Oleanane, Ursane and Lupane Triterpenoid Acids Inhibit Biofilm Formation by P. aeruginosa PAO1

**Figure 6:**
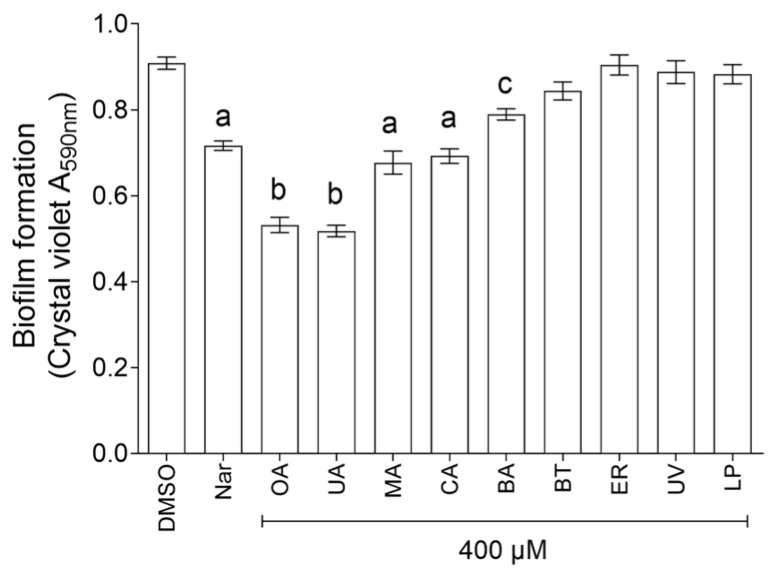
Anti-biofilm effects of oleanane, ursane and lupane triterpenoids in *P. aeruginosa* PAO1. Quantification of biofilm formation by *P. aeruginosa* PAO1 grown in minimal medium after static incubation at 37 °C for 24 h. Biofilm formation was quantified using crystal violet staining and measured by A_590nm_. All triterpenoids [(erythrodiol (ER), oleanolic acid (OA), maslinic acid (MA), uvaol (UV), ursolic acid (UA), corosolic acid (CA), lupeol (LP), betulin (BT) and betulinic acids (BA)]) were applied at 400 µM and naringenin at 4000 µM was used as a positive control. DMSO-treated cultures were used as controls and experiments were conducted following five technical replicates and three independent biological replicates. Different letters (a, b, c) above the bars indicate data that are statistically different from DMSO and from each other according to a one-way ANOVA with Tukey’s multiple comparison test (*p* < 0.01) and error bars represent the standard errors of the means.

To further investigate the anti-virulence potential of these triterpenoids, their effect on *P. aeruginosa* PAO1 biofilm formation was assessed at 400 µM after 24 h of bacterial growth. Notably, significant inhibition of biofilm formation was observed when the PAO1 strain was grown in the presence of OA, UA, MA, CA and BA as compared to the DMSO control (Figure 6) with inhibition rates of 45%, 40%, 30%, 32% and 15% of inhibition, respectively. In contrast, LP, BT, UV and ER did not affect PAO1 biofilm formation under the same conditions.

### 2.4. Ursolic and Oleanolic Acid Disorganize the P. aeruginosa PAO1 Biofilm Architecture

**Figure 7:**
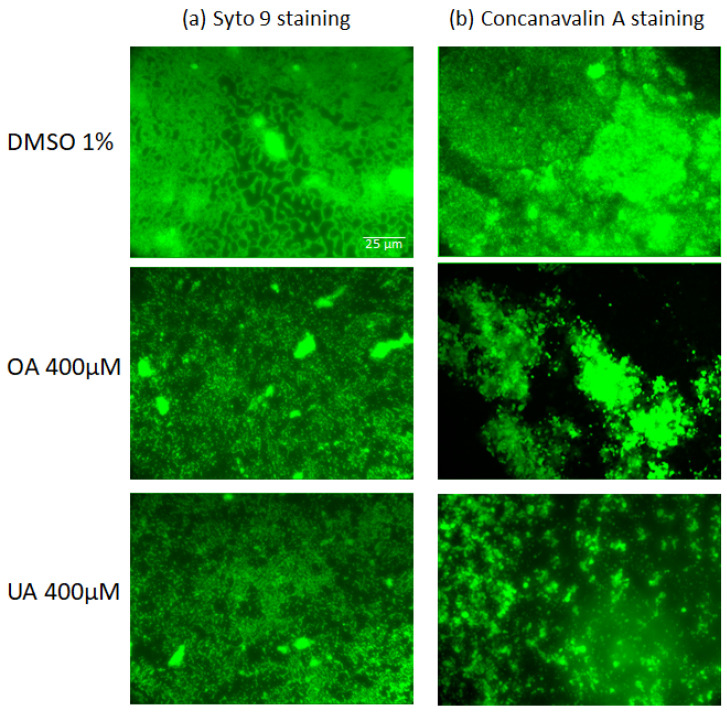
PAO1 biofilm phenotypes as affected by control DMSO, oleanolic acid (OA), or ursolic acid (UA). (**a**) Fluorescence microscopy images of PAO1 cells incubated statically at 37 °C for 24 h. Cells were visualized after staining with SYTO-9. (**b**) Fluorescence microscopy images of exopolysaccharides produced by PAO1 cells incubated for 24 h and visualized after staining with concanavalin A FITC. OA and UA were tested at 400 µM. False-coloured images were captured using an inverted fluorescence microscope, Leica DM IRE2(400× magnification) from Leica Microsystems GmbH (Wetzlar, Germany), and assembled using Adobe Photoshop.

**Figure 8:**
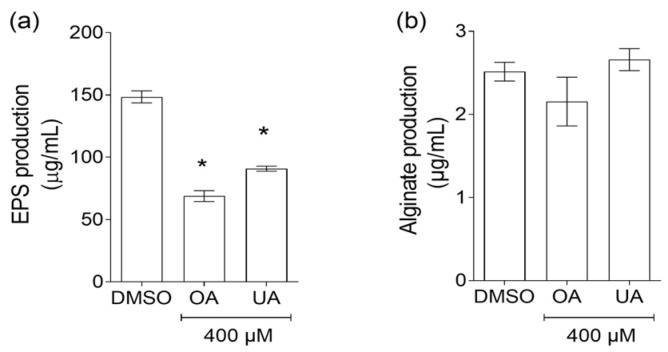
Effect of oleanolic acid (OA) and ursolic acid (UA) at 400 μM, on extracellular polysaccharides production by *P. aeruginosa* PAO1. (**a**) Quantification of total extracellular polysaccharides was achieved using a phenol-sulfuric acid method and expressed in μg/mL with reference to a glucose standard. (**b**) Quantification of alginate was performed using a carbazole method and expressed in μg/mL with reference to a sodium alginate standard. For both experiments, bacterial density was assessed at A_600nm_. All experiments were conducted following five technical replicates and three independent biological replicates. Asterisks indicate samples that are significantly different from the control DMSO (*p* < 0.01), and error bars represent the standard errors of the means.

As OA and UA exhibit the strongest anti-biofilm activity, their impact on the biofilm phenotype of *P. aeruginosa* PAO1 was evaluated. Fluorescence microscopy with Syto-9 staining indicated that, after 24 h of growth under static conditions, the control *P. aeruginosa* PAO1 culture (DMSO) forms a thick and homogenous biofilm layer on coverslips with well-connected colonies, interspaced with uncolonized regions (Figure 7a). In contrast, UA- and OA-treated *P. aeruginosa* PAO1 cells present a reduction in compact cell clusters and microcolonies confluence (Figure 7a). The presence of EPS was visually compared among PAO1 treated with OA or UA and DMSO by using concanavalin-A lectin FITC staining, which allows detecting polysaccharides containing α-D-mannose and α-D-glucose based on its affinity to terminal residue α-D-mannosyl and α-D-glucosyl [30]. As shown in Figure 7b, terminal α-D-mannose/α-D-glucose polysaccharides were uniformly distributed across the coverslip surface under the DMSO control condition. In contrast, OA- and UA-treated samples showed irregular polysaccharide aggregates, suggesting that parts of *P. aeruginosa* PAO1 colonies were not properly embedded in exopolysaccharide matrices. Consistently, the quantification of extracellular polysaccharides produced over 24 h of growth revealed a significant reduction in OA- and UA-treated PAO1 cells (Figure 8a; 62 and 48% inhibition, respectively). In contrast, the production of the specific acidic polysaccharide alginate was not impacted under the same conditions (Figure 8b).

## 3. Discussion

In this study, the anti-QS and anti-biofilm activities of a selection of pentacyclic triterpenoids were investigated. These compounds are derived from the oxidation at C-28 and/or hydroxyl addition at the C-2 position of α-amyrin (ursane-type precursor) that generate uvaol, ursolic acid and corosolic acid, β-amyrin (oleanane-type precursor) that generate erythrodiol, oleanolic acid and maslinic acid) and lupeol (lupane-type precursor) that generate betulin and betulinic acid. Some of these precursors and their derivatives (acetate forms) have previously been shown to inhibit biofilm formation in various pathogenic bacteria. Our team previously reported that α-amyrin at 200 µM, isolated from *P. rotundifolium*, inhibits biofilm formation by *P. aeruginosa* PAO1 at 200 µM without affecting bacterial viability or QS circuitry [26]. Moreover, β-amyrin acetate from *Vernonia auriculifera* Hiern. significantly decreased the adhesion of *Staphylococcus aureus* (ATCC 43300), *K. pneumonia* and *Enterococcus faecium* at 1 mM (sub-MIC concentrations) [24]. By contrast, exposure to lupeol acetate from *Ficus sansibarica* Warb. subsp. *sansibarica* (Moraceae) at 4.3 mM resulted in a significant increase in biofilm formation in *E. coli* ATCC 29922, *E. coli* ATCC 35218, *S. aureus* ATCC 29213 and *S. aureus* ATCC 43300 [31].

These reports are consistent with the present study in which most of the ursane- and oleanane-type triterpenoids exhibited significant anti-virulence and anti-biofilm activities against *P. aeruginosa* PAO1. In contrast, among the lupane-type triterpenoids, only BA showed a weak inhibitory effect on *lasB* and *rhlA* gene expression (17% and 14%, respectively) as well as on biofilm formation (15%). Notably, in the PAO1 model, all tested triterpenoid alcohols (UV, ER and LP) failed to inhibit QS-related gene expression or biofilm formation (Figure 6). Taken together, these results suggest two plausible structure-activity relationships: (*i*) the presence of an isopropenyl-cyclopentane, a substituted E-ring, in the lupane skeleton, instead of a dimethyl-cyclohexane, may hinder key interaction with as yet unidentified biological targets; and (*ii*) oxidation of the C-28 alcohol moiety into a carboxylic group drastically enhances the anti-virulence properties of triterpenoids.

Intriguingly, a recent study demonstrated that BA at 200 µg/mL (≈440 µM) strongly inhibits biofilm formation by *Acinetobacter baumanii* ATCC19606 and *P. aeruginosa* PA14 (88% and 80% of inhibition, respectively) [32]. As we previously noticed, Rajkumari et al. [23] reported that both BA and BT significantly attenuated the production of QS-regulated virulence factors and biofilm formation in *P. aeruginosa* PAO1 (57% and 32% of inhibition, respectively) at a sub-lethal concentration (≈275 µM), which diverges from the results obtained in the present study. These discrepancies suggest that the inhibition mechanisms may be strain-dependent (PA14 vs. PAO1), genome-dependent (given that phenotypic variability among PAO1 isolates has been noticed by Klockgether et al. [33]) and/or culture-conditions-dependent. The latter factor warrants further investigation as it could be relevant for potential translational applications. Indeed, *P. aeruginosa* strains grown under conditions promoting motility (media supplemented with glutamate and succinate as carbon sources or citrate minimal medium) form flat and uniform biofilms, whereas low motility conditions (glucose as carbon source) generated mushroom-shaped biofilms [11,34]. Accordingly, differences in growth conditions, including inoculum size (initial A_600nm_ of inoculum of 0.04 [23] instead of 0.14 in this study) and culture medium (tryptone soya broth [23] versus minimal medium broth containing glucose as sole carbon source in this study), may influence the kinetics of biofilm development and structuration in *P. aeruginosa*, which may affect its responses to biofilm perturbators. Indeed, a glucose-based minimal medium induces a nutrient stress that rapidly promotes bacterial adhesion, followed by development of a well-structured biofilm, whereas a rich complex medium allows bacteria to maintain a planktonic lifestyle, leading to weak surface-adhesion of cells and thick but less structured cell aggregates likely to be more easily disrupted [35]. Such divergences denote the difficulty for interpreting anti-biofilm properties and highlight the importance of standardized experimental protocol, that closely mimic infective conditions, including extreme carbon and nitrogen limitation, with sparse and diverse nutrients (e.g., host proteins, mucin, lipids) rather than a continuous, high-energy carbon source [36], low oxygen availability, limited specific nutrients such as iron, host cell interaction, … [37].

Previously, we have shown that a C-3 coumarate ester of oleanolic aldehyde (OALC), isolated from *Dalbergia trichocarpa* Baker, exerts anti-QS and anti-biofilm activities against *P. aeruginosa* PAO1. This compound inhibits PAO1 biofilm formation and maintenance by 44% at 200 µM, compared with the DMSO control, and reduces the expression of the *las* and *rhl* QS systems by more than 50% [38]. This dual effect of OALC on both QS regulation and biofilm formation was proposed to result from coumaroyl and pentacyclic triterpenoid groups, respectively. Although oleanolic aldehydes have not been tested here, the present results suggest that the oleanane triterpenoid structure can confer the anti-biofilm activity without impacting on QS circuitry. In the same line, UA and the 3*β*-O-cis-p-coumaroyl-20*β*-hydroxy-12-ursen-28-oic acid (a C-3 coumarate ester of 20*β*-hydroxyursolic acid), both isolated from *Diospyros dendo* Welw, have been reported to strongly inhibit *P. aeruginosa* PAO1 biofilm formation [25,39]. These compiled data indicate that C-3 coumarate esterification of oleanane or ursane triterpenoid acids enhances anti-biofilm activity, whereas hydroxylation at the C-2 position [e.g., CA (2α-hydroxyursolic acid) and MA (2α-hydroxyoleanolic acid)] leads to a slight reduction in anti-biofilm activity as observed in Figure 6. Thus, for increased efficiency, esterification at the C-3 position, particularly with a well-known anti-QS moiety, should be considered to design semi-synthetic derivative anti-biofilm compounds based on the triterpenoid scaffold.

At this stage, it is premature to propose a mechanism of action of these anti-biofilm triterpenoids. However, as they do not impact the *las* and *rhl* system while disrupting exopolysaccharide production, it is possible that they interfere with the pool regulation of c-di-GMP, which is implicated in the regulation of exopolysaccharide production and the switch from planktonic to biofilm lifestyle modes through the control of bacterial motility [40]. High c-di-GMP levels activate exopolysaccharide biosynthesis, whereas low c-di-GMP levels favour flagella formation and planktonic growth. Accordingly, further investigations are needed to explore the effects of triterpenoid acids on the c-di-GMP pool and their impact on motility. Moreover, since OA and UA did not affect alginate production (Figure 8), it will be important to evaluate their effects on Psl and Pel polysaccharides production to confirm the reduction in total exopolysaccharide content observed in treated PAO1.

To conclude, pentacyclic triterpenoid acids represent promising chemical backbones for the development of compounds targeting *P. aeruginosa* biofilm formation. Moreover, given their strong anti-biofilm activities, the relevance of using OA as a positive control for anti-biofilm assays is confirmed, and UA could be proposed as another positive control option, in place of naringenin, which exhibits only 20% inhibition of biofilm formation at 4 mM concentration (Figure 6).

## 4. Materials and Methods

### 4.1. Bacterial Strains, Plasmids and Culture Conditions

The *P. aeruginosa* PAO1 strain and its derivatives used in this work have been previously reported by Vandeputte et al. [27] and by Rasamiravaka et al. [28]. The strains were grown (37 °C, agitation 175 rpm) in LB-MOPS broth (Luria–Bertani–morpholinepropane sulfonic acid; 50 mM, pH 7) for the PAO1 wildtype strain and supplemented with carbenicillin (300 μg/mL) for its derivatives. QS-related promoter-*lacZ* fusions (*lasB-lacZ*, *lasI-lacZ*, *lasR-lacZ*, *rhlA-lacZ*, *rhlI-lacZ*, *rhlR-lacZ*) and QS-independent promoter-*lacZ* fusions (*aceA*-*lacZ*) were used.

### 4.2. Chemicals

All pentacyclic triterpenoids tested in this study (Figure 2) were purchased from Sigma-Aldrich (Buchs, Switzerland), dissolved in 100% DMSO and used at 100–800 µM (final concentration, resulting in 1% DMSO). These include three oleanane triterpenoids: ER [(3β)-olean-12-ene-3,28-diol], OA [(3β)-hydroxyolean-12-en-28-oic acid] and MA [(2α, 3β)-dihydroxyolean-12-en-28-oic acid]; three ursane triterpenoids: UV [(3β)-urs-12-ene-3,28-diol], UA [(3β)-hydroxyurs-12-en-28-oic acid] and CA [(2α, 3β)-dihydroxyurs-12-en-28-oic acid]; and three lupane triterpenoids: LP [(3β) -lup-20(29)-en-3-ol], BT [(3β)-lup-20(29)-ene-3,28-diol] and BA [(3β)-hydroxylup-20(29)-en-28-oic acid]. Naringenin, used as an anti-QS and anti-biofilm positive control [27,28], was purchased from Sigma-Aldrich, dissolved in 100% DMSO and tested at 4000 µM (final concentration, resulting in 1% DMSO).

### 4.3. Gene Expression and β-Galactosidase Measurements

To quantify gene expression of QS-dependent (*lasB* and *rhlA*) and QS-regulatory (*lasR/I* and *rhlR/I*), the β-galactosidase activity was measured using *O*-nitrophenyl-β-D-galactopyranoside as substrate [27,41]. Following an overnight growth in liquid LB-MOPS-Carbenicillin at 37 °C and 175 rpm, PAO1 reporter strains were washed twice in fresh LB broth medium and resuspended in liquid LB-MOPS-Carbenicillin. Fifty µl of adjusted PAO1 reporter strains inoculums, in order to achieve an initial A_600nm_ of culture comprised between 0.020 and 0.025, were incubated (37 °C with 175 rpm agitation) for 18 h in 940 µL LB-MOPS-Carbenicillin supplemented with 10 μL of triterpenoids dissolved in DMSO (100 to 800 μM) or 10 μL of DMSO (1%, *v*/*v*) or the flavanone naringenin (4000 µM, known as QS inhibitor [27]). After incubation, the bacterial population density was estimated by spectrophotometry (A_600nm_), bacterial viability was counted by the CFU experiment as described by Sieuwerts et al. [42] and gene expression by the β-galactosidase assay [27]. A QS-independent gene, i.e., the isocitrate lyase encoding *aceA* expression, was also evaluated for global gene transcription monitoring.

### 4.4. Biofilm Visualization and Quantification

*P. aeruginosa* PAO1 was grown overnight in LB medium at 37 °C with agitation (175 rpm). After growth, the bacterial suspension was centrifuged (4500 rpm) for 5 min and the recovered bacterial pellet was diluted with Biofilm Broth (BB), a minimal medium as described by Khalilzadeh et al. [43]. In a 24-well plate (Greiner Bio-One’s CELLSTAR^®^, Frickenhausen, Germany), 25 μL of the diluted culture was added to 470 μL of BB (initial A_600nm_ of culture comprised between 0.14 and 0.16) supplemented with 5 μL of DMSO (1%, *v*/*v*) or triterpenoids (800 μM) or naringenin (4000 µM). After a static incubation for 24 h at 37 °C, planktonic bacteria were discarded from the plate and adherent biofilms were gently washed with water (2 mL) and fixed with 2 mL of methanol (99%) for 15 min. Prior to the crystal violet staining step, the methanol was discarded and completely removed from the plate by evaporation. Crystal violet (0.1% in water) was then added to fill each well. After 30 min of incubation, crystal violet was then discarded, and stained biofilms were gently washed three times with 1 mL of water. To solubilize the fixed dye, acetic acid (33% in water) was added and the absorbance of the solution was measured at 590 nm with a SpectraMaxM2 device (Molecular Devices, Wokingham, UK).

The biofilm formation by PAO1 cells was also evaluated in glass coverslip cultures by fluorescence microscopy in order to evaluate the effects of these triterpenoids on the biofilm structure of *P. aeruginosa*. Experiments follow the same culture conditions as described above. After 24 h incubation, the growth medium was replaced by 500 mL of SYTO 9 (diluted 400-fold in BB medium) or concanavaline-A lectin FITC (diluted in BB medium for a 100 µg/mL final concentration) solutions. Biofilms were incubated away from light for 15 min and PAO1 cells were visualized using a Leica DM IRE2 inverted fluorescence microscope coupled to a CCD camera (Leica DC350 FX, Leica Microsystems GmbH, Wetzlar, Germany) and equipped with FITC.

### 4.5. Total Extracellular Polysaccharides and Alginate Quantification

As described by Rasamiravaka et al. [37], extracellular polysaccharides were extracted using ethanol and quantified with the phenol-sulfuric acid method, whereas extraction and quantification of alginate were achieved by cetylpyridinium chloride precipitation and a modified carbazole-based method that detects uronic acids, respectively.

### 4.6. Statistics

All experiments were conducted following five technical replicates and three independent biological replicates. Student’s *t*-tests or one-way ANOVA with Tukey’s multiple comparison test were used to evaluate the statistical significance (*p*-value < 0.01) of data via GraphPad Prism 8 software.

## Data Availability

Data are contained within the article.

## Supplementary Materials

The following supporting information can be downloaded at: https://www.mdpi.com/article/10.3390/antibiotics15060623/s1, Table S1: Effect of the three triterpenoids type (oleanane, ursane and lupane) on *P. aeruginosa* PAO1 growth; Figure S1: Effect of the three triterpenoids type (oleanane, ursane and lupane) on *lasB*, *rhlA* and *aceA* genes expression in *P. aeruginosa* PAO1: (**a**) Effect of triterpenoids on *lasB* expression following 18 h of growth. (**b**) Effect of triterpenoids on *rhlA* expression following 18 h of growth. (**c**) Effect of triterpenoids on *aceA* expression following 18 h of growth. All triterpenoids (erythrodiol (ER), oleanolic acid (OA), maslinic acid (MA), Uvaol (UV), Ursolic acid (UA), Corosolic acid (CA), Lupeol (LP), Betulin (BT) and Betulinic acid (BA)) were used at 800 µM and naringenin at 4000 µM was used as positive control. Gene expression was measured as the β-galactosidase activity of the *lacZ* gene fusions and expressed in Miller units. Error bars represent the standard errors of the means; all experiments were performed in quintuplicate with three independent assays. Asterisks indicate samples that are significantly different from the DMSO (*p* < 0.01).

## Abbreviations

The following abbreviations are used in this manuscript:

BA	Betulinic acid
BB	Biofilm Broth
BT	Betulin
CA	Corosolic acid
c-di-GMP	Bis-(3′-5′)-cyclic dimeric guanosine monophosphate
DMSO	Dimethyl sulfoxide
ER	Erythrodiol
LB-MOPS	Luria–Bertani–morpholinepropane sulfonic acid
LP	Lupeol
MA	Maslinic acid
OA	Oleanolic acid
UA	Ursolic acid
UV	Uvaol
