# Supplementary material for: Pentacyclic Triterpenoid Acids Inhibit the Expression of Quorum Sensing-Related Virulence Factors and the Formation of Biofilm in Pseudomonas aeruginosa PAO1
Source: Antibiotics (Basel). 2026 Jun 20;15(6):623. doi: 10.3390/antibiotics15060623 (PMC13295455; doi:10.3390/antibiotics15060623)
Supplement: Supplementary file 1 [file antibiotics-15-00623-s001.zip › Figure S1.pdf]

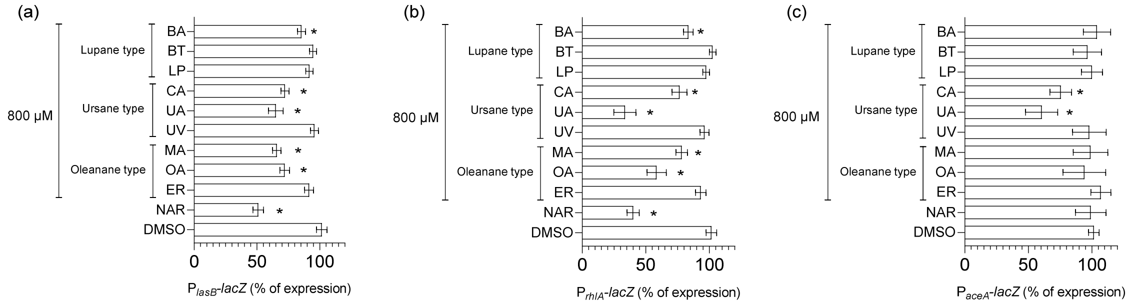

Figure S1. Effect of the three triterpenoids type (oleanane, ursane and lupane) on *lasB, rhlA and aceA* genes expression in *P. aeruginosa* PAO1: (**a**) Effect of triterpenoids on *lasB* expression following 18 hours of growth. (**b**) Effect of triterpenoids on *rhlA* expression following 18 hours of growth. (**c**) Effect of triterpenoids on *aceA* expression following 18 hours of growth. All triterpenoids (erythrodiol (ER), oleanolic acid (OA), maslinic acid (MA), Uvaol (UV), Ursolic acid (UA), Corosolic acid (CA), Lupeol (LP), Betulin (BT) and Betulinic acid (BA)) were used at 800 µM and naringenin at 4000 µM was used as positive control. Gene expression was measured as the β-galactosidase activity of the *lacZ* gene fusions and expressed in Miller units. Error bars represent the standard errors of the means; all experiments were performed in quintuplicate with three independent assays. Asterisks indicate samples that are significantly different from the DMSO (p < 0.01).
