# Supplementary material for: Pentacyclic Triterpenoid Acids Inhibit the Expression of Quorum Sensing-Related Virulence Factors and the Formation of Biofilm in Pseudomonas aeruginosa PAO1
Source: Antibiotics (Basel). 2026 Jun 20;15(6):623. doi: 10.3390/antibiotics15060623 (PMC13295455; doi:10.3390/antibiotics15060623)
Supplement: Supplementary file 1 [file antibiotics-15-00623-s001.zip › Table S1.pdf]

Table S1. Effect of the three triterpenoids type (oleanane, ursane and lupane) on *P. aeruginosa* PAO1 growth.

Culture condition	Growth (UFC/mL)	Turbidity (A600nm)*
PAO1+DMSO1%	$4.9x10^{11}$	1.46
PAO1+Nar(4000µM)	$8.0x10^{11}$	1.51
PAO1+ER(800µM)	$6.2x10^{11}$	1.43
PAO1+OA(800µM)	$5.8x10^{11}$	1.51
PAO1+MA(800µM)	$4.9x10^{11}$	1.49
PAO1+UV(800µM)	$7.1x10^{11}$	1.47
PAO1+UA(800µM)	$6.2x10^{11}$	1.44
PAO1+CA(800µM)	$4.9x10^{11}$	1.48
PAO1+LP(800µM)	$7.1x10^{11}$	1.51
PAO1+BT(800µM)	$5.8x10^{11}$	1.50
PAO1+BA(800µM)	$4.9x10^{11}$	1.51

Naringenin (Nar), dimethylsulfoxide (DMSO), erythrodiol (ER), oleanolic acid (OA) and maslinic acid (MA), three ursanes, uvaol (UV), ursolic acid (UA) and corosolic acid (CA), and three lupanes, lupeol (LP), betulin (BT) and betulinic acid (BA).

*Turbidity measurement of bacterial suspension have been realized prior CFU experiment.
